# Integrated metastate functional connectivity networks predict change in symptom severity in clinical high risk for psychosis

**DOI:** 10.1002/hbm.25235

**Published:** 2020-10-13

**Authors:** George Gifford, Nicolas Crossley, Sarah Morgan, Matthew J Kempton, Paola Dazzan, Gemma Modinos, Matilda Azis, Carly Samson, Ilaria Bonoldi, Beverly Quinn, Sophie E Smart, Mathilde Antoniades, Matthijs G Bossong, Matthew R Broome, Jesus Perez, Oliver D Howes, James M Stone, Paul Allen, Anthony A Grace, Philip McGuire

**Affiliations:** ^1^ Department of Psychosis Studies Institute of Psychiatry, Psychology and Neuroscience, King's College London London UK; ^2^ Department of Psychiatry, School of Medicine Pontificia Universidad Católica de Chile Santiago Chile; ^3^ Department of Psychiatry University of Cambridge Cambridge UK; ^4^ The Alan Turing Institute London UK; ^5^ Department of Psychological Medicine Institute of Psychiatry, Psychology and Neuroscience, King's College London London UK; ^6^ South London and Maudsley NHS Trust Maudsley Hospital London UK; ^7^ Department of Neuroimaging, Institute of Psychiatry, Psychology and Neuroscience King's College London London UK; ^8^ CAMEO Early Intervention in Psychosis Service Cambridgeshire and Peterborough NHS Foundation Trust Cambridge UK; ^9^ MRC Centre for Neuropsychiatric Genetics and Genomics, Division of Psychological Medicine and Clinical Neurosciences, School of Medicine Cardiff University Cardiff UK; ^10^ Department of Psychiatry, Icahn Medical School Mt Sinai Hospital New York New York USA; ^11^ Department of Psychiatry, UMC Utrecht Brain Center Utrecht University Utrecht The Netherlands; ^12^ Institute for Mental Health School of Psychology, University of Birmingham Birmingham UK; ^13^ Department of Psychology University of Roehampton London UK; ^14^ Departments of Neuroscience, Psychiatry and Psychology University of Pittsburgh Pittsburgh Pennsylvania USA

**Keywords:** cartographic profile, clinical high‐risk for psychosis, network analysis, network based statistics, network integration, task fMRI

## Abstract

The ability to identify biomarkers of psychosis risk is essential in defining effective preventive measures to potentially circumvent the transition to psychosis. Using samples of people at clinical high risk for psychosis (CHR) and Healthy controls (HC) who were administered a task fMRI paradigm, we used a framework for labelling time windows of fMRI scans as ‘integrated’ FC networks to provide a granular representation of functional connectivity (FC). Periods of integration were defined using the ‘cartographic profile’ of time windows and k‐means clustering, and sub‐network discovery was carried out using Network Based Statistics (NBS). There were no network differences between CHR and HC groups. Within the CHR group, using integrated FC networks, we identified a sub‐network negatively associated with longitudinal changes in the severity of psychotic symptoms. This sub‐network comprised brain areas implicated in bottom‐up sensory processing and in integration with motor control, suggesting it may be related to the demands of the fMRI task. These data suggest that extracting integrated FC networks may be useful in the investigation of biomarkers of psychosis risk.

## Graphical abstract

A framework for extracting epochs of integrated functional connectivity was used to test network differences between healthy control and clinical high‐risk (CHR) groups, as well as associations with longitudinal changes in symptom and functioning scores in CHR participants. A sub‐network suggestive of bottom‐up sensory processing was found to be associated with changes in positive psychotic symptoms in the CHR group. We suggest this to be a useful approach in searching for network‐based biomarkers of psychosis risk.

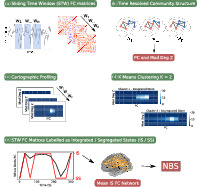

## 1 INTRODUCTION

A key challenge in the clinical management of people at clinical high risk (CHR) for psychosis is that it is difficult to predict whether their presenting symptoms will improve, persist, or progress to a frank psychotic disorder, and if their overall level of functioning will improve or deteriorate (Fusar‐Poli et al., 2012; Simon et al., 2013). This has led to a search for biological measures that might help clinicians to predict clinical outcomes in this group (Gifford et al., 2017). The present study aimed to achieve this by exploring the association of novel functional connectivity (FC) based biomarkers with longitudinal changes in psychosis symptoms and functioning.

The CHR state is associated with subjective impairments in cognitive function, which are often described as Basic Symptoms (Huber & Gross, 1989). These include difficulties in dividing attention between sensory modalities, and focusing attention on non‐salient visual stimuli (Schultze‐Lutter, Klosterkötter, Picker, Steinmeyer, & Ruhrmann, 2007). Neuropsychological assessments in CHR subjects indicate that this state is also associated with objective cognitive impairments across multiple domains (Fusar‐Poli et al., 2012), including attention and vigilance (Zheng et al., 2018).

Neuroimaging studies have shown a range of alterations in functional connectivity (FC) in CHR participants (Allen et al., 2010; Crossley et al., 2009; Schmidt et al., 2014; Winton‐Brown et al., 2017). Such studies have shown alterations in ‘static’ FC by modelling connectivity profiles over the entirety of a scan. It has been suggested that in healthy individuals however, that the brain switches between periods of network integration and segregation, and that the state of network integration is associated with periods of attention (Shine, Koyejo, & Poldrack, 2016) and task performance (Shine, Bissett, et al., 2016). Recent work has exhibited intermittent alterations in FC in CHR subjects (Du et al., 2018), raising the possibility that switching between network segregation to integration may be perturbed in this group. Such a deficit might underlie the deficits in attention and sensory processing in CHR subjects described above. The present study aimed to address this issue by using fMRI to examine FC in CHR subjects within periods of integration (acting as a proxy for periods of high attention/vigilance) whilst they were performing a cognitive task.

We computed the ‘cartographic profile’ (CP) (Guimera, R., & Amaral, L. A. N., 2005; Shine, Bissett, et al., 2016; Shine, Koyejo, & Poldrack, 2016) during a novelty salience fMRI paradigm, which is a method that can be used to mark integrated and segregated ‘metastates’ during a scan. Network Based Statistics (NBS) (Zalesky, Fornito, & Bullmore, 2010) were then used to search for network differences during integrated metastates between groups of CHR subjects and controls. The CHR samples were then followed up clinically to determine their clinical and functional outcomes. This allowed us to investigate whether the disruption of FC networks derived from integrated metastates is related to subsequent outcomes in CHR participants. Our first hypothesis was that FC networks derived from integrated metastates would be altered in CHR subjects relative to controls. We then tested the hypothesis that this would be associated with clinical and functional outcomes in these subjects at follow‐up.

## 2 METHODS

### 2.1 Participants

One hundred and sixteen participants consented to the present study. Four CHR participants were excluded from analysis because they did not fully complete the three task fMRI runs, and one was excluded because a macroscopic abnormality was detected on MRI scanning. A further 30 participants (23 CHR/7 HC) were removed from the analysis due to the stringent motion criteria (see pre‐processing section and Supplementary Material Section 2), leaving 81 participants (24 HC/57 CHR).

All participants were studied using fMRI at the Centre for Neuroimaging Studies, King's College London. The study received ethical approval from the National Health Service UK Research Ethics Committee and all participants gave written informed consent. CHR participants were scanned when they presented to one of four specialist clinical services: Outreach and Support in South London (OASIS) (South London and Maudsley NHS Trust), the West London Early Intervention Service, Cambridge Early Onset (CAMEO) (Cambridge and Peterborough NHS Trust), and the Coventry and Warwick Warwickshire Partnership NHS Trust. HC participants were recruited from the same geographical areas as the CHR sample through local advertisements.

All participants were between 18 and 35 years of age. General recruitment exclusion criteria included any MRI contraindications (e.g., pregnancy, claustrophobia) or history of neurological illness, substance dependency, or diagnosis of psychotic disorders, according to DSM 4 criteria (Bell, 1994). HC participants were excluded if they had a personal or familial history of psychiatric/neurological illness. All participants reported no illicit substance use in the past week and no alcohol use in the 24 hour period before scanning.

Participant demographic information is shown in Table 1. There were significant group differences in age, and in treatment with antipsychotic and antidepressant medications. Age and medication use were therefore controlled for in group comparisons. Past and present medication use were merged into two binary variables (one variable for antidepressant use and one for antipsychotic use) to be used as a covariate (1 = past or present medication use, 0 = no past or present medication use).

**TABLE 1:** Demographics including N, median age, sex, ethnicity, handedness, mean years in education, mean IQ, medication, mean GAF symptom and function scores at baseline, mean PANSS positive and negative symptom scores at baseline

	Baseline group comparison sample			Follow up PANSS sample		Follow up GAF function sample	
	HC	CHR	Significance test		Significance test		Significance test
N	24.00	57.00		27.00		31.00	
Median age (IQR)	25.58 (4.28)	22.82 (3.75)	*p* = .005	23.00 (5.50)	*p* = .780	22.00 (4.50)	*p* = .854
Male N (%)	11.00 (45.83%)	36.00 (63.16%)	*p* = .232	17.00 (62.96)	*p* = 1.000	18.00 (58.06)	*p* = .811
Right handed *N* (%)	22 (91.67%)	48 (84.21%)	*p* = .492	24.00 (88.89%)	*p* = .743	27.00 (87.10%)	*p* = 1.000
Years in education (*SD*)	16.13 (3.32)	14.72 (2.31)	*p* = .073	14.59 (2.59)	*p* = .834	14.38 (2.29)	*p* = .527
IQ (*SD*)	105.19 (11.36)	105.57 (11.17)	*p* = .896	109.60 (9.43)	*p* = .102	106.45 (11.02)	*p* = .733
Medication *N* (%)							
Past antipsychotic use	0.00 (0.00%)	3.00 (5.26%)	*p* < .001	6.00 (22.22%)	*p* = .767	5.00 (16.13%)	*p* = 1.000
Past antidepressant use	0.00 (0.00%)	9.00 (15.79%)	*p* < .001	11.00 (40.74%)	*p* = .818	12.00 (38.71%)	*p* = .659
Antipsychotic at time of MRI	0.00 (0.00%)	10.00 (17.54%)	*p* = .029	4.00 (14.81%)	*p* = 1.000	3.00 (9.68%)	*p* = 1.000
Antidepressant at time of MRI	1.00 (4.17%)	25.00 (43.86%)	*p* < .001		*p* = 1.000	11.00 (35.48%)	*p* = 1.000
Antipsychotic at follow up				4.00 (14.81%)	*p* = .460	3 (9.68%)	*p* = 1.000
Antidepressant at follow up				10.00 (37.04%)	*p* = .110	9 (29.03%)	*p* = .589
Mean GAF function (*SD*)	91.00 (7.50)	60.00 (19.00)		57.11 (10.32)	*p* = .325	56.81 (11.10)	*p* = .270
Mean PANSS total		55.87 (14.67)		56.96 (13.43)	*p* = .740	56.87 (14.97)	*p* = .769
Mean PANSS positive (*SD*)		14.00 (5.00)		13.70 (3.72)	*p* = .961	13.93 (4.14)	*p* = .767
Mean PANSS negative (*SD*)		11.00 (6.00)		12.67 (4.46)	*p* = .857	12.50 (4.20)	*p* = .978
Median days to follow up (IQR)				511.03 (299.95)		527.26 (310.84)	
Mean change in PANSS total scores (*SD*)				5.70 (21.20)		4.83 (21.44)	
Mean change in PANSS positive scores (*SD*)				0.30 (5.55)		0.13 (5.90)	
Mean change in PANSS negative scores (*SD*)				1.37 (5.67)		0.96 (5.52)	
Mean change in GAF Disability score (*SD*)				3.00 (20.94)		2.74 (20.35)	

*Note:* For follow‐up cohorts mean change in PANSS total, positive, and negative symptoms scores, GAF functioning scores, and median number of days to follow‐up are shown. Significance tests show difference between HC and CHR groups for baseline comparisons, and difference between CHR baseline and follow‐up samples (those with follow up PANSS/GAF scores). Significance test used for Age: Mann–Whitney U, Sex: Chi‐squared, Handedness: Fischer's Exact test, Years in Education: T test, IQ: T test., Medication: Fischer's Exact Test, GAF Function: T test, PANSS total/negative/positive: T test.

Abbreviations: *SD*, standard deviation, IQR, inter quartile range.

A proportion of the CHR sample underwent follow up assessments at a median of 458 days (*N* = 46, IQR = 376). Follow‐up time was highly positively skewed. The GAF and PANSS were used to assess the level of functioning and psychotic symptom severity at follow‐up, in 31 and 27 subjects, respectively. Transition to psychosis and persistence of the CHR state was assessed using the Comprehensive Assessment of At‐Risk Mental States (CAARMS; Yung et al., 2002). Eight CHR participants had developed a psychotic disorder during follow‐up, whilst 8 were in remission from the CHR state. Because the number of transitions and remissions were limited, only functional and symptom level outcomes were investigated in the analyses. Continuous symptom and functioning scores were used to maximise statistical power. Demographics of the sub‐samples with follow‐up data are shown in Table 1. Loss to follow‐up did not appear to bias the follow‐up cohorts in any measure (Table 1).

### 2.2 Clinical scales

The CHR state was diagnosed using the CAARMS. Pre‐morbid IQ was measured using the National Adult Reading Test (Nelson & Willison, 1991), variation in functioning was measured the functional subscale from the Global Assessment of Functioning scale (Karterud, Pedersen, Løvdahl, & Friis, 1998), and psychotic symptoms were assessed using the positive and negative syndrome scale (PANSS) (Kay, Flszbein, & Opfer, 1987).

### 2.3 Code sharing

The present study used Matlab scripts made available for modelling Cartographic Profiles (Shine, Koyejo, & Poldrack, 2016) Multiplication of Temporal Derivatives (Shine et al., 2015) and multilayer community structure (Jutla, Jeub, & Mucha, 2011; Mucha, Richardson, Macon, Porter, & Onnela, 2010). NBS was performed using the available software (Zalesky et al., 2010). Post hoc tests and statistics were performed using tailor‐made scripts in R 3.5.1 and Python 3.6.8.

### 2.4 Task fMRI paradigm

Each participant was administered a novelty salience task derived from that described by Bunzeck and Düzel (2006). Each block consisted of 80 standard images, 10 neutral oddballs, 10 novel oddballs, and 10 target images. Participants were asked to respond to target images with a button press. Images were presented for 100 ms followed by a fixation cross, with an inter‐image interval of 2,700 ms. Each run lasted for 6 min (179 volumes, TR 2 s). Reaction time and error rate from this task indicates performance on target button presses and not instances of novelty stimulus presentation. Data from the present study using this task has previously been published using Dynamic Causal Modelling (Modinos et al., 2020).

### 2.5 Pre‐processing

Functional MRI scans were pre‐processed using a standard fMRIPREP pipeline (Esteban et al., 2019). A full description of the pre‐processing used is given in the Supplementary Materials Section 1. A high pass cut off of 128 s was included in this pipeline (discrete cosine basis during CompCor extraction) (Behzadi, Restom, Liau, & Liu, 2007), which is equivalent to bandpass filtering that had been used in previous research using the same task paradigm (Bunzeck & Düzel, 2006; Modinos et al., 2020).

Head motion has a problematic effect on FC‐derived network measures (Satterthwaite et al., 2012; Van Dijk, Sabuncu, & Buckner, 2012). In the present study, participants were excluded from analysis if more than 25% of volumes contained a framewise displacement (FD) > 0.25 mm or DVARS >4%, or they had a mean FD > 0.2. This was done in addition to an ICA‐based removal of motion artefacts (Pruim et al., 2015). In the final cohort mean FC remained significantly different between the two groups (mean FD (*SD*): HC = 0.10 (0.03), CHR = 0.11 (0.03); *t* (47.68) = 2.05, *p* = .046) and so mean FD was controlled for in group comparisons and in associations with changes in functioning/symptom scores. As we were using dynamic FC methods, volume censoring was not performed, in order to preserve the temporal structure of scans. A full description of motion correction procedures is given in the Supplementary Materials Section 2.

### 2.6 Region of interest definition

Time‐series were averaged within cortical/subcortical regions of interest using 5 mm spheres centred at 264 coordinate locations (cortical and subcortical) from Power et al. (2011). To negate the effects of inflated connectivity due to task events, HRF‐convolved task event data were regressed out of the time‐series, the same process used in another study using the cartographic profile with task fMRI data (Shine, Bissett, et al., 2016). Nodes with high signal drop out were removed from the analysis, leaving 204 of the original 264 regions. An area of signal drop out was defined as an area with a mean time‐series signal intensity z score (across nodes) less than or equal to −1.64 in any subject, corresponding to outliers <5th percentile.

### 2.7 Multiplication of temporal derivatives

In order to provide an estimate of FC that is robust against task‐based inflation of connectivity between regions, multiplication of temporal derivatives (MTD) was used (Shine et al., 2015). To calculate the first temporal derivative for node i at time‐point t (*dt*_*it*_) of a time series (*ts*), the bold intensity at time point t − 1 was subtracted from that at time point t (dt_it_ = ts_it_ − ts_it − 1_). Each node was given a vector of dts across the time series (t − 1), which were then normalised by the *SD* across that vector (σ). A node x node MTD matrix for each time point was calculated by multiplying each dt for each pair of nodes ij (Shine et al., 2015). We used non‐overlapping windows of 15 TR (30s), which produced a total of 33 windows (11 windows including 15 volumes in each of 3 runs).

### 2.8 Cartographic profile

To define whether time windows represented integrated or segregated metastates, a procedure for defining the ‘cartographic profile’ (CP) was used (Guimera et al., 2005; Shine, Bissett, et al., 2016). The CP is a 2D histogram of the frequency distribution of two graph metrics, the Participation Coefficient (PC) and the Module Degree Z‐score (MDZ). Both the PC and MDZ rely on a network being segregated into distinct communities, as the PC is a measure of between‐community connectivity, whilst the MDZ a measure of within‐community connectivity (for formula see Guimera et al., 2005). The PC and MDZ were determined using the Brain Connectivity Toolbox (http://www.brain-connectivity-toolbox.net/). To find the community structure of all time windows, a multilayer modularity maximisation algorithm was used (γ/ɷ = 1) (Jutla et al., 2011; Mucha et al., 2010). Once this had been found for each time window, the frequency distributions of node wise PC and MDZ were used to create one CP for each time window. A study of how methodological choices affected the distribution of the CP in this study is described in the Supplementary Materials Section 3. In addition, an exploration of the effects of window‐length, window offset, and CP resolution was performed, which supported the parameters used in the current study in the context of extracting periods of highly integrated FC (Supplementary Materials Section 5). Such methodological choices may not apply to other forms of dynamic functional connectivity analysis where, for instance, higher temporal resolution may be beneficial.

### 2.9 Creating one integrated network for each participant

**FIGURE 1:**
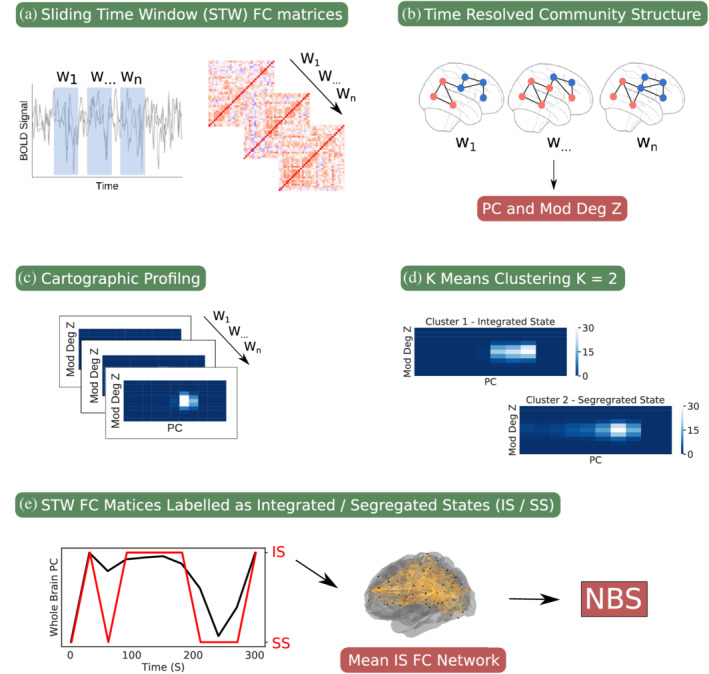
The analysis pipeline involved the following steps: (a) A sliding time window (STW) was used to extract a sequence of functional connectivity (FC) matrices. (b) Multilayer community detection was used to find the time resolved community structure of FC matrices. (c) The cartographic profile (CP) of each FC matrix was found using graph metrics (module degree z‐score/participation coefficient) derived from the time resolved community structure. (d) K means clustering was used to cluster CPs into two states (Integrated/Segregated). (e) FC matrices labelled as Integrated States were averaged to create one mean Integrated State FC matrix for each participant to use in NBS within/between group comparisons

We aimed to create one integrated network for each participant, to use in within‐ and between‐group comparisons. This involved taking the CP of each time window, using a k‐means clustering algorithm to label time‐windowed CPs as integrated/segregated (within participant), then using these labels to create an average integrated FC network for each participant. Participant integrated FC networks were then used in within‐ and between‐group comparisons. This procedure is illustrated in Figure 1.

A deviation in our methodology from that in previous studies (Shine, Bissett, et al., 2016; Shine, Koyejo, & Poldrack, 2016) is that instead of using CPs with a dimensionality of 100 × 100, we used CP dimensions of 10 × 10. This was done to reduce the dimensionality of the feature space in the clustering procedure, and because it appeared to give more distinct CP distributions between integrated/segregated metastates (see Supplementary Materials Section 3). In addition, because of the non‐deterministic behaviour of the multilayer community detection algorithm, the procedure for finding an integrated FC network was repeated 100 times. Because the study included three runs, the integrated FC network used for within‐ and between‐group comparisons were computed as the mean across the three runs and 100 repetitions. The CP procedure uses K means clustering with K = 2. Validation of the choice of clusters is shown in Supplementary Materials Section 4.

### 2.10 Between/within group comparisons: Network Based Statistics

Network Based Statistics (NBS; Zalesky et al., 2010) were used to identify sub‐networks that differed in terms of connectivity strength between CHR and HC groups, and that were associated with longitudinal changes in GAF and PANSS scores in the CHR group (change = follow up score — baseline score). This was done firstly using integrated FC networks and secondly, as a point of reference, using ‘static’ FC networks, computed as an average of all time window FC matrices for each participant.

NBS works firstly by applying a test statistic threshold to provide a set of above threshold sub‐networks and then performing a permutation test on the maximum sub‐network size expected to give the significance of these sub‐networks (Zalesky et al., 2010). For between group comparisons (HC vs. CHR) test statistic thresholds were defined using a one‐way ANCOVA with group status as the independent variable and with the following covariates: age, mean FD, history of antipsychotic use, and history of antidepressant use. For within CHR group associations with change in GAF and PANSS scores, a multiple linear regression was used, with the covariates of sex, age, mean FD, history of antipsychotic use, history of antidepressant use, and the number of days between the baseline scan and follow up visit.

It is typical to test the NBS procedure across a range of initial thresholds (Nelson, Bassett, Camchong, Bullmore, & Lim, 2017; Zalesky et al., 2010). In the present study, a range of F threshold from 6 to 28 was used. For any sub‐networks found to be significantly associated with change in symptom/functioning scores, we also tested whether baseline scores would yield similar results, to confirm that results were specific to a change in scores. In addition, we tested whether excluding participants taking antipsychotic medication at the time of the MRI scan or at follow up would result in similar findings.

## 3 RESULTS

### 3.1 Task performance

There was no difference between the two groups in hit rate (percentage of correct target hits) (median (IQR): HC = 100 (0), CHR = 100 (3.33), U = 625, *p* = .446) or reaction time (ms) (means (*SD*): HC = 556.83 (83.31), CHR = 566.35 (104.08), t (53.65) = 0.43, *p* = .666). Because the majority of participants had a hit accuracy of 100% (suggesting a ceiling effect), only reaction time was used as a measure of task performance.

### 3.2 Cartographic profile: Group differences

There were no significant group differences in the median number of communities in each run (U = 536.50, *p* = .128), or the mean time spent in an integrated metastate (t [49.72] = 1.15, *p* = .255). The mean number of metastate switches was significantly higher in the HC group (t [52.51] = −2.26, *p* = .028) (Table 2), however not if taking into account multiple comparisons correction.

**TABLE 2:** Significance tests, medians/means, and *SD*/interquartile range (IQR) for the proportion (%) of time spent in an integrated metastate, and the number of metastate switches

	HC		CHR		Significance test	*p*
Median number of communities per run (IQR)	3.10	0.51	3.04	0.61	U = 536.50	.128
Mean % time in an integrated metastate (*SD*)	61.05	9.23	63.76	10.68	T (49.72) = 1.15	.255
Mean number of metastate switches (IQR)	3.25	0.60	2.89	0.73	T (52.51) = −2.26	.028

Across the whole sample (CHR and HC) metastate switching was not correlated with reaction time (R_s_ = −0.18, *p* = .100), or with time spent in an integrated metastate (R_s_ = 0.21, *p* = .060). However, ‘flexibility’, a measure based on the proportion of times nodes switch allegiance between multilayer communities (Bassett et al., 2011), was negatively correlated with reaction time (R_s_ = −0.44, *p* < .001), suggesting it was a better reflection of brain dynamics in relation to task performance. Flexibility was not significantly different between the two groups (Mean [*SD*]: CHR = 0.30 [1.17], HC = 0.37 [1.01], *t* [44.09] = −1.33, *p* = .192).

### 3.3 Between/within group comparisons (NBS)

There were no significant group differences in sub‐networks found using either static or integrated connectivity matrices (one‐way analysis of variance [ANCOVA]; FWE *p* ≤ .025; 5,000 permutations).

Within the CHR group, when longitudinal changes in PANSS positive scores were analysed using integrated FC matrices, one sub‐network was found to be significant (F thresholds 19–27 at FWE *p* ≤ .025). A similar sub‐network was found to be associated with the change in PANSS positive scores when excluding those taking antipsychotic medication at the time of MRI and at follow up, though only at *p* ≤ .05 (Supplementary Materials Section 6).

**FIGURE 2:**
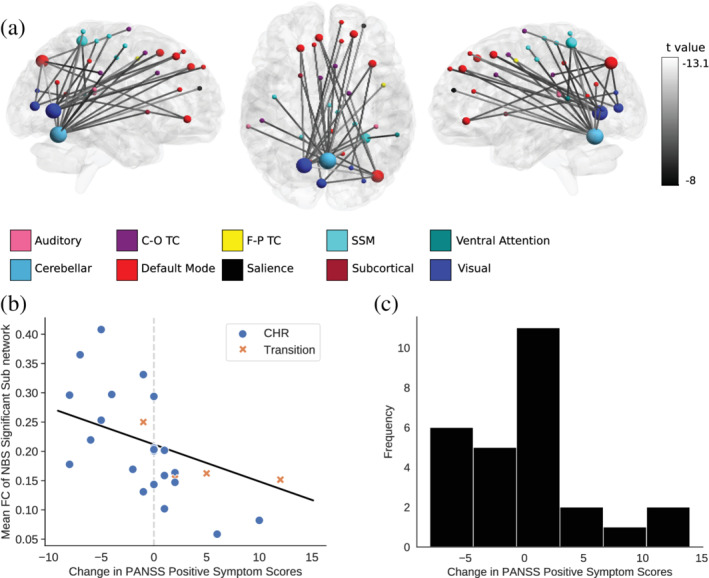
(a) Nodes and edges of a sub‐network found to be significantly associated with changes in PANSS positive symptom scores in a sample of CHR participants. T values and edge colour from equivalent multiple linear regressions. Edges are shown in grayscale with the lighter greys corresponding to more extreme T values. Node size represents nodal degree. (b) Scatter‐plot and simple regression line (Pearson's R = −0.42) for mean connectivity of the whole NBS significant sub‐network with change in PANSS positive symptom score. Regression line shown in black. Dashed line shows no change in PANSS positive symptom scores. (c) Distribution of change in PANSS positive symptom scores in the sub‐sample of CHR participants used in within group comparisons. C‐O TC, cingulo‐opercular task control; F‐P TC, fronto‐parietal task control; SSM, somatosensory‐motor

An arbitrary NBS statistic threshold (F = 19, *p* = .023) was chosen for interpretation (Zalesky et al., 2010). The sub‐network mainly included occipital‐frontal and cerebellar‐frontal connections, including highly connected nodes in the right V area of the cerebellum (MNI XYZ: 1, −62, −18) and the intercalcarine cortex (MNI XYZ: 20, 66, 2). Nodes mostly included frontal and parietal areas of the default mode network, and sensory (visual, auditory, sensory‐somatomotor) and cingulo‐opercular task control networks. T values from identical multiple linear regressions used in the NBS procedure, but computed on single connections, suggested that all connections reflected a negative association with change in PANSS positive scores. T values here were used post hoc to infer the direction of the relationship, and were not indicated as being individually significant from the NBS procedure. Using identical multiple regression models it was also possible to find the mean Cohen's f^2^ to indicate the effect size of models with the response variable (PANSS positive symptom scores). This was done across all brain network edges (upper triangular of the matrix) giving a mean Cohen's f^2^ of 0.46 (*SD* = 0.30). Restricting this to edges of the sub‐network found in the NBS procedure resulted in a mean Cohen's f^2^ of 1.84 (*SD* = .59). Additionally, we examined the correlation between the mean FC of the whole sub‐network and changes in PANSS positive scores, in order to test whether the results were due to participants who became more or less symptomatic in terms of the positive relationship with FC. This indicated that the CHR sample included both subjects who became more symptomatic and subjects who became less symptomatic, and that both contributed to the negative relationship with FC in the significant sub‐network. Results are shown in Figure 2 and Table 3.

We also tested whether using the baseline PANSS positive symptom scores (as opposed to the change in these scores) as the dependent variable would produce similar results. No significant sub‐networks were found, suggesting the findings were specific to longitudinal changes in symptomatology. In addition, the procedure was repeated excluding those who were taking antipsychotic medication at the time of MRI, which suggested the found association with PANSS positive symptom scores to be independent of current antipsychotic medication use.
There were no sub‐networks associated with longitudinal changes in GAF or PANSS negative scores, using either static or highly integrated connectivity matrices (multiple linear regression; FWE *p* ≤ .025; 5,000 permutations). When changes in PANSS positive symptom scores were analysed using static connectivity matrices there were no significant sub‐networks.
A supplementary analysis was performed in order to also explore regional variation in integration and segregation (Supplementary Materials Section 7). This was done using the Recruitment (R_i_) and Integration Coefficients (I_i_) (Mattar, Cole, Thompson‐Schill, & Bassett, 2015). Whilst there were no regional integration/segregation values (I_i_/R_i_) found to be significantly different between groups, or associated with change in positive symptom scores in CHR participants, there was a whole brain trend for higher I_i_ in the CHR group. Notably, using the CP procedure CHR participants spent more time in an integrated metastate (Table 2) and whole brain I_i_ across the whole sample was strongly correlated with time spent in an integrated metastate (*R* = .76).

**TABLE 3:** Regions, MNI Coordinates (X, Y, Z), resting state network affiliation (Power et al., 2011), and degree (within sub‐network) of nodes in a sub‐network found to be significantly associated with longitudinal change in PANSS positive scores in CHR participants, using an NBS procedure (multiple linear regression; FWE *p* ≤ .025; 5,000 permutations) on FC matrices derived from integrated metastates

Region	X	Y	Z	Resting state network	Degree
Precentral gyrus	0	−15	47	Sensory‐somatomotor	1
Precentral gyrus	−7	−21	65	Sensory‐somatomotor	1
Precentral gyrus	44	−8	57	Sensory‐somatomotor	1
Superior parietal lobule	−29	−43	61	Sensory‐somatomotor	4
Superior parietal lobule	22	−42	69	Sensory‐somatomotor	1
Postcentral gyrus	−21	−31	61	Sensory‐somatomotor	1
Anterior supramarginal gyrus	54	−28	34	Cingulo‐opercular task control	1
Superior frontal gyrus	−16	−5	71	Cingulo‐opercular task control	1
Juxtapositional lobule	7	8	51	Cingulo‐opercular task control	1
Anterior cingulate gyrus	−5	18	34	Cingulo‐opercular task control	1
Posterior superior temporal gyrus	65	−33	20	Auditory	1
Parietal operculum cortex	−38	−33	17	Auditory	1
Superior lateral occipital cortex	−39	−75	44	Default mode	6
Precuneous cortex	−11	−56	16	Default mode	1
Precuneous cortex	15	−63	26	Default mode	1
Posterior cingulate gyrus	−2	−37	44	Default mode	1
Superior frontal gyrus	23	33	48	Default mode	2
Superior frontal gyrus	−10	39	52	Default mode	1
Superior frontal gyrus	−35	20	51	Default mode	2
Frontal pole	−10	55	39	Default mode	1
Frontal pole	−20	45	39	Default mode	2
Paracingulate gyrus	8	42	−5	Default mode	2
Anterior cingulate gyrus	12	36	20	Default mode	1
Lateral occipital cortex	−28	−79	19	Visual	1
Intercalcarine cortex	20	−66	2	Visual	11
Intracalcarine cortex	−18	−68	5	Visual	1
Intracalcarine cortex	6	−81	6	Visual	3
Precentral gyrus	−44	2	46	Fronto‐parietal task control	1
Frontal pole	−28	52	21	Salience	1
Putamen	23	10	1	Subcortical	1
Planum temporale	−55	−40	14	Ventral attention	1
Cerebellum (right V)	1	−62	−18	Cerebellar	15

## 4 DISCUSSION

The present study has demonstrated how a process for extracting periods of high brain network integration from an fMRI scan can be used to search for novel network biomarkers in a CHR population. The main finding was that a sub‐network identified using this approach was negatively associated with changes in PANSS positive symptom scores. In contrast, analyses using static FC networks did not yield significant results.

### 4.1 Dynamic functional connectivity network organisation

CHR participants were suggested to have less frequent switching between integrated and segregated metastates, though not when controlling for multiple comparisons. Less switching between integrated and segregated metastates in the CHR group could reflect relatively less dynamic functional brain organisation in the CHR group. However, metastate switching was not related to mean RT, whereas flexibility, a measure that has been related to task performance in multiple studies (Bassett et al., 2011; Braun et al., 2015; Pedersen, Zalesky, Omidvarnia, & Jackson, 2018; Telesford et al., 2016), did not differ between groups and was negatively correlated with mean RT, suggesting it was a better index of dynamic brain organisation related to task performance. In addition, several studies of flexibility have suggested that schizophrenia is associated with higher, not lower, dynamic network switching (Braun et al., 2016; Gifford et al., 2020). We summarise that this study does not suggest less dynamic functional brain organisation in CHR populations.

Time spent in an integrated state was also not correlated with any measures of task performance, in contrast to data from a previous study (Shine, Bissett, et al., 2016). However, levels of integration and segregation are dependent on task demands (Shine, Bissett, et al., 2016), and the network dynamics in the present study may be specific to the cognitive demands of the task we used. The high accuracy rate for most participants in the present study suggests the task used required low cognitive effort. Similar to Shine, Bissett, et al. (2016), we found that participants spent more time in an integrated metastate, suggesting some validity of the present methodology.

### 4.2 Discovery of psychosis‐related sub‐networks

A sub‐network was found to be negatively associated with changes in PANSS positive scores, although only when using FC matrices derived from integrated (as opposed to static) metastates. This suggests FC patterns during periods of network integration may provide a useful framework for identifying biomarkers of symptomatic outcome. We suggest that using the framework of separating FC profiles into integrated and segregated metastates may produce a more granular signal than those found using an entire scan.

The present study attempted to use task fMRI data in a manner that disregarded all task events, whilst evaluating underlying functional activity. However, the sub‐network associated with change in PANSS positive symptom scores could be related to task demands, specifically the integration of bottom‐up sensory processing with required task motor behaviour. A central node in this network covered the right V area of the cerebellum. This area is implicated in task‐related hand movements (King, Hernandez‐Castillo, Poldrack, Ivry, & Diedrichsen, 2019; Stoodley & Schmahmann, 2009, 2010). Moreover, another central node was found in the intracalcarine cortex, which plays a key role in processing visual information and is thought to be involved in a bottom‐up process of attributing salience to visual information (Koene & Zhaoping, 2007; Li, 2002; Zhang & Li, 2012). Furthermore, connections between visual areas and the parietal lobe could be interpreted as part of the ‘dorsal visual stream’, which is involved in planning action based on visual information (Galletti & Fattori, 2018; Rizzolatti & Matelli, 2003). In addition, default mode nodes within this sub‐network included the precuneus, a centrally important node in the default mode network (Fransson & Marrelec, 2008), and implicated in visuomotor learning (Kawashima, Roland, & O'Sullivan, 1995; Wenderoth, Debaere, Sunaert, & Swinnen, 2005). Finally, the sub‐network included several areas of the cingulo‐opercular task control network, thought to be involved in goal‐directed behaviour that remains stable across task sets (Dosenbach et al., 2007).

A relationship of the findings to task demands does not necessarily mean that they are not relevant to psychotic symptoms. For example, the severity of positive psychotic symptoms has previously been associated with dysfunction in visual motion integration (Carter et al., 2017) and ‘soft’ neurological signs (Buchanan & Heinrichs, 1989), which are thought to reflect sensory integration abnormalities, are more frequent in CHR subjects (Lawrie et al., 2001) and patients with psychosis (Dazzan & Murray, 2002) than controls. A previous study in CHR participants also associated longitudinal changes in positive symptoms with dynamic FC (computed as the *SD* of FC) of the superior temporal gyrus, visual cortex, and somatosensory cortex (Pelletier‐Baldelli, Andrews‐Hanna, & Mittal, 2018).

The stronger FC of the sub‐network negatively associated with positive symptoms might reflect compensatory effort in visual sensory motor integration, which was protective against the later development of positive symptoms. This would be consistent with the notion that network integration reflects periods of high cost brain activation, as shown by associations with attention/vigilance (Shine, Bissett, et al., 2016). The sub‐network associated with positive symptoms involved highly spatially distributed connections, suggesting a high wiring cost, though alternatively could reflect less efficient brain circuitry.

### 4.3 Strengths and limitations

Strengths of the present study include the use of a relatively large sample of CHR subjects for an fMRI study, combined with the presence of longitudinal outcome data. The logistical difficulties of recruiting, scanning, and following up very large samples would require multi‐site collaboration, which would introduce the confound of site effects (Gifford et al., 2017). Most of the CHR sample (83.87%) were naïve to antipsychotic medication at the time of scanning. Moreover, we controlled for medication exposure by entering it as a covariate in the analyses. Excluding those taking antipsychotic medication at the time of MRI or at follow up also resulted in similar results. Therefore, although antipsychotic medication might influence FC (Bolding et al., 2012; Dandash et al., 2014; Fornito et al., 2013; Nejad, Ebdrup, Glenthøj, & Siebner, 2012), the results from the present study are not suggested to reflect the effects of antipsychotic medication. A caveat however, is that the amount and duration of medication use between time‐points was not available to account for historical medication use between visits. Though medication use at the time of assessment was accounted for, medication use between visits may have influenced results.

A key limitation in the present study is that the negative relationship between positive psychotic symptoms and the identified sub‐network appeared to be related both to participant increase and decrease in symptoms. Whilst the findings may suggest possible neurobiology protective of psychosis development, it therefore presents no clinical usage. Additionally, the use of task‐based fMRI data adds complexity to the replication and interpretation of the sub‐network we found to be associated with changes in psychotic symptoms. The cartographic profile has previously been compared across resting state and various task paradigms, showing increased integration in integrated states with more cognitively demanding tasks (Shine, Bissett, et al., 2016). This suggests there may be a benefit in using a task paradigm to extract highly integrated brain states. Future studies may wish to use more cognitively demanding tasks to extract periods of high integration in exploring psychosis risk.

The results of the present study may be dependent on multiple hyper‐parameters (Supplementary Materials Section 2), as well as the tuning of the NBS procedure according to the statistical threshold for suprathreshold links (Zalesky et al., 2010). A high number of impactful methodological choices is a barrier to reliability (Simmons, Nelson, & Simonsohn, 2011; Wicherts et al., 2016). It should be noted that choice of the statistic threshold in NBS does not affect the family wise error rate, rather the sensitivity to sub‐network discovery (Zalesky et al., 2010).

Classifying windows based on global cartographic profiles may not take into account regional variation in integration/segregation. A supplementary analysis explored dynamic regional variation in integration/segregation (Supplementary Materials Section 7), suggesting no localised dynamic integration/segregation signals to significantly associate with CHR status or change in symptom scores. As such, we may suggest the whole brain CP procedure to be preferable in searching for clinically relevant neural substrates such as those associated with changes in positive psychosis symptoms in the current study, as compared with mass‐univariate testing of regional signals.

An assumption of this study is that the brain switches between whole brain epochs of integration and segregation. Although a k = 2 solution was suggested to be appropriate in the current study (Supplementary Materials Section 4) and in previous similar studies (Fukushima et al., 2018; Shine, Bissett, et al., 2016; Shine et al., 2015; Shine, Koyejo, & Poldrack, 2016), it is important to highlight that other methodological approaches have resulted in higher numbers of distinct intermittent brain states when clustering time windowed FC profiles (Allen et al., 2014; Damaraju et al., 2014).

## 5 CONCLUSIONS

The use of methods for defining intermittent states of integration and segregation (Guimera et al., 2005; Shine, Bissett, et al., 2016; Shine, Koyejo, & Poldrack, 2016) may provide a useful framework for future fMRI studies searching for biomarkers in psychosis.

## CONFLICT OF INTERESTS

O. D. H. has received investigator‐initiated research funding from and/or participated in advisory/speaker meetings organised by Astra‐Zeneca, Autifony, BMS, Eli Lilly, Heptares, Jansenn, Lundbeck, Lyden‐Delta, Otsuka, Servier, Sunovion, Rand and Roche. AAG receives consulting fees from Johnson & Johnson, Lundbeck, Pfizer, Takeda, Alkermes, Otsuka, Lilly, Roche, Asubio. The other authors declare no competing financial interests.

## Supporting information


**Appendix** **S1:** Supporting information

## Data Availability

At the time the data was collected it was not routine for participants to be asked for their consent to share data publicly, so this permission was not obtained. Whilst we are in favour of data being open access, no supporting data is available for this study.
