# Supplementary material for: Integrated metastate functional connectivity networks predict change in symptom severity in clinical high risk for psychosis
Source: Hum Brain Mapp. 2020 Oct 13;42(2):439–51. doi: 10.1002/hbm.25235 (PMC7775992; doi:10.1002/hbm.25235)
Supplement: Supplementary file 1 — Appendix S1: Supporting information [file HBM-42-439-s001.docx]

1. Supplementary Materials - HBM-20-0525
2. Corresponding Author: George Gifford [george.gifford@kcl.ac.uk](mailto:george.gifford@kcl.ac.uk)

Table of Contents

[Section 1 - FMRI Pre-processing (FMRIPREP Boilerplate) 1](#__RefHeading___Toc16794_3332057006)

[Section 2 - Movement and Functional Connectivity 2](#__RefHeading___Toc16796_3332057006)

[Section 3 - Investigating the Effects of Methodological Choices on Cartographic Profile Distribution 4](#__RefHeading___Toc16798_3332057006)

[Section 4 - K Mean Clustering – Finding the Optimal Number of Clusters 7](#__RefHeading___Toc16800_3332057006)

[Section 5 - Investigating the Effect Of Window Offset, Length, and Cartographic Profile Resolution 9](#__RefHeading___Toc16802_3332057006)

[Section 6 - Investigating the Effect Of Medication Use on Sub-networks Associated with Change in PANSS Positive Symptom Scores 11](#__RefHeading___Toc16804_3332057006)

[Section 7 - Regional Variation in Integration and Segregation 12](#__RefHeading___Toc378_3313028695)

[References 14](#__RefHeading___Toc16806_3332057006)

## FMRI Pre-processing (FMRIPREP Boilerplate)

1. Results included in this manuscript come from preprocessing performed using FMRIPREP version 1.2.6-1 (Esteban et al., 2019), a Nipype (Gorgolewski et al., 2011) based tool. Each T1w (T1-weighted) volume was corrected for INU (intensity non-uniformity) using N4BiasFieldCorrection v2.1.0 (Tustison et al., 2010) and skull-stripped using antsBrainExtraction.sh v2.1.0 (using the OASIS template). Spatial normalization to the ICBM 152 Nonlinear Asymmetrical template version 2009c (Fonov et al., 2009) was performed through nonlinear registration with the antsRegistration tool of ANTs v2.1.0 (Avants et al., 2008), using brain-extracted versions of both T1w volume and template. Brain tissue segmentation of cerebrospinal fluid (CSF), white-matter (WM) and gray-matter (GM) was performed on the brain-extracted T1w using fast (Zhang et al., 2001).
2. Functional data was slice time corrected using 3dTshift from AFNI v16.2.07 (Cox, 1996) and motion corrected using mcflirt (Jenkinson et al., 2002) (FSL v5.0.9). This was followed by co-registration to the corresponding T1w using boundary-based registration (Greve & Fischl, 2009) with 9 degrees of freedom, using flirt (FSL). Motion correcting transformations, BOLD-to-T1w transformation and T1w-to-template (MNI) warp were concatenated and applied in a single step using antsApplyTransforms (ANTs v2.1.0) using Lanczos interpolation.
3. Physiological noise regressors were extracted applying CompCor (Behzadi et al., 2007). Principal components were estimated for the two CompCor variants: temporal (tCompCor) and anatomical (aCompCor). A mask to exclude signal with cortical origin was obtained by eroding the brain mask, ensuring it only contained subcortical structures. Six tCompCor components were then calculated including only the top 5% variable voxels within that subcortical mask. For aCompCor, six components were calculated within the intersection of the subcortical mask and the union of CSF and WM masks calculated in T1w space, after their projection to the native space of each functional run. Frame-wise displacement (Power et al., 2014) was calculated for each functional run using the implementation of Nipype. ICA-based Automatic Removal Of Motion Artifacts (AROMA) was used to generate aggressive noise regressors as well as to create a variant of data that is non-aggressively denoised (Pruim et al., 2015).
4. Many internal operations of FMRIPREP use Nilearn (Abraham et al., 2014), principally within the BOLD-processing workflow. For more details of the pipeline see <https://fmriprep.readthedocs.io/en/latest/workflows.html>.

## Movement and Functional Connectivity

In the current study stringent quality control thresholds for head motion were applied. Participants were removed if more than 25% of volumes contained a framewise displacement (FD) > 0.25 mm or DVARS > 4%. It also appeared that the distribution of mean FD was positively skewed for CHR participants and so participants were additionally removed if they had a mean FD > 0.2 over the three runs (Figure 1).


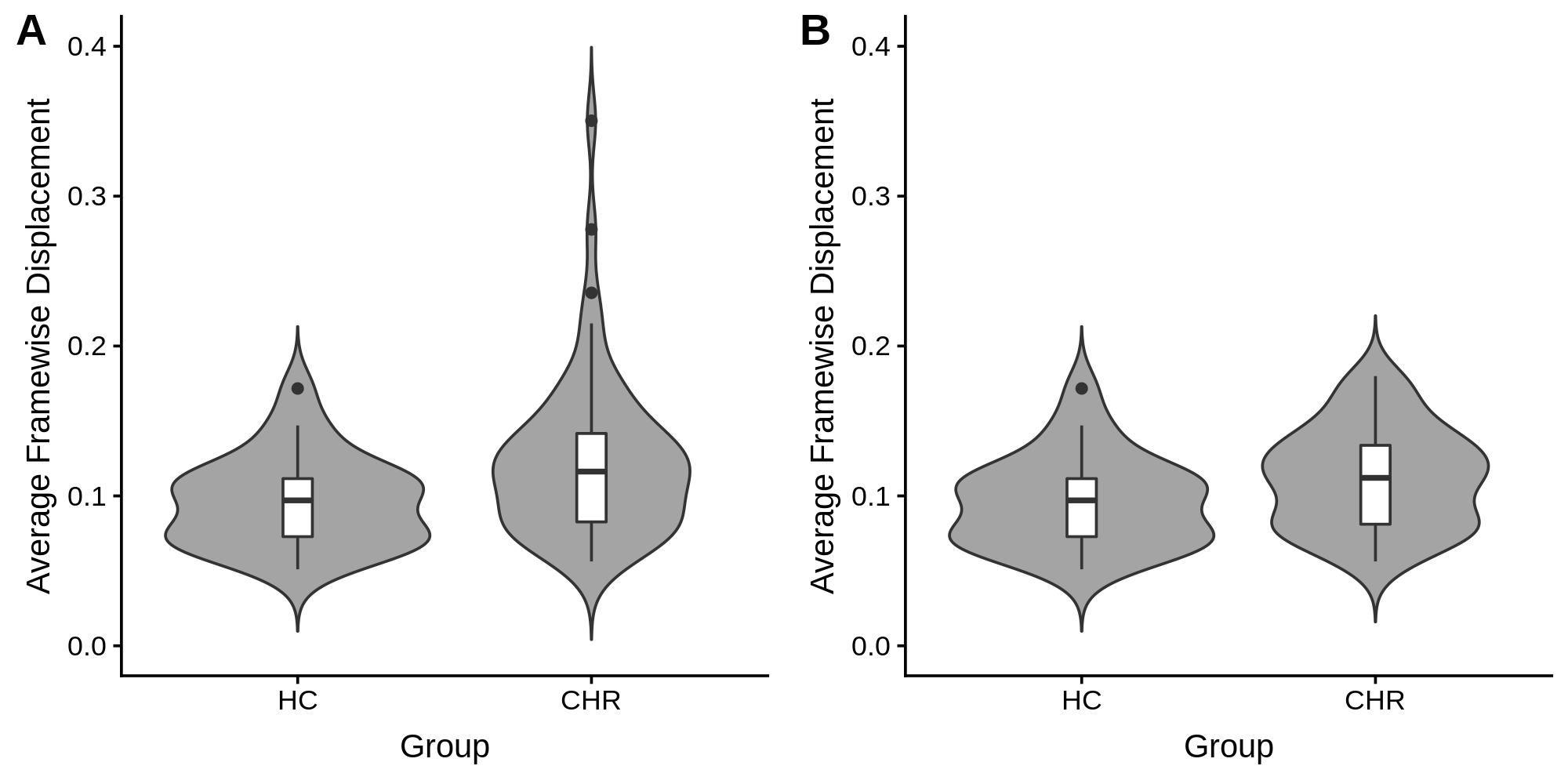


*Figure 1: Violin plots of mean frame-wise displacement over three task runs. A) Distribution over the whole sample. B) Distribution over the sample excluding those with a mean FD > 0.2 mm.*

Though removing high movement participants increased the similarity between the two cohorts in terms of mean FD (figure 1), there was still a significant difference between the two cohorts (t (47.68) = 2.05, p = 0.046). Satterthwaite plots (edge-wise correlation between functional connections and motion vs Euclidean distance of node pairs) (Satterthwaite et al., 2012) also suggested a weak, but present, relationship between mean FD and functional connectivity (FC) for more distant connections (Figure 2). For the above reasons, mean FD was controlled for in within / between group comparisons.


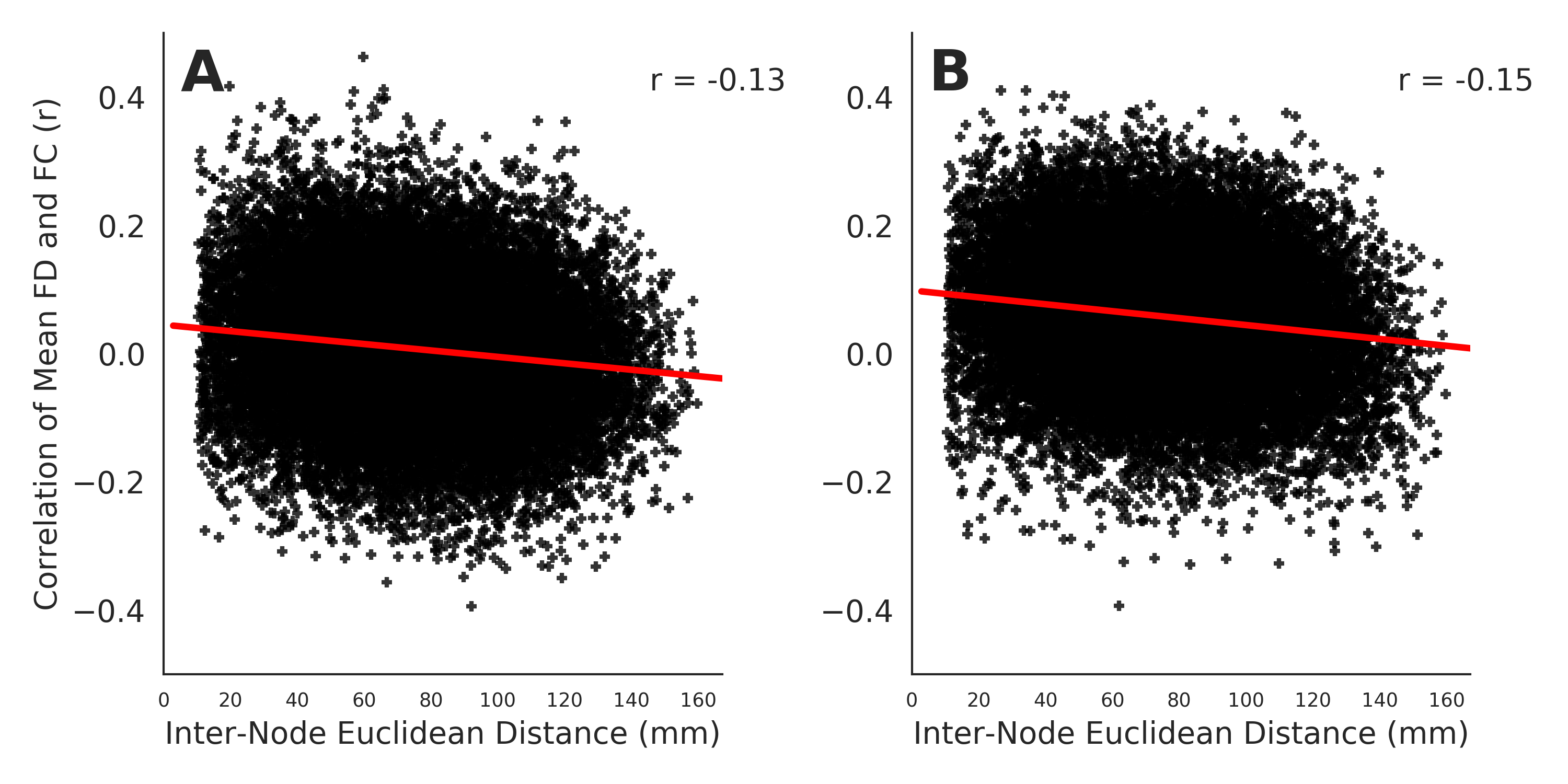


Figure 2: Scatter-plot and regression line for inter-node Euclidean distance and correlations between mean Framewise Displacement (FD) and Functional Connectivity (FC) (Multiplication of Temporal Derivatives), across all participants. r = Pearson’s correlation coefficient. A) Static FC computed as the mean across all time windows. B) Integrated FC computed as the mean across integrated states.

## Investigating the Effects of Methodological Choices on Cartographic Profile Distribution

We used the distributions of the cartographic profile (CP) of integrated and segregated states (mean across all participants) to infer whether the procedure had worked correctly, with the assumption that more spatially distinct distributions reflected better separation of the two states. Though a similar distribution of integrated / segregated CPs to previous studies was not found (Fukushima et al., 2018; Shine, Bissett, et al., 2016; Shine, Koyejo, et al., 2016) it was possible to see what methodological choices resulted in more distinct distributions.


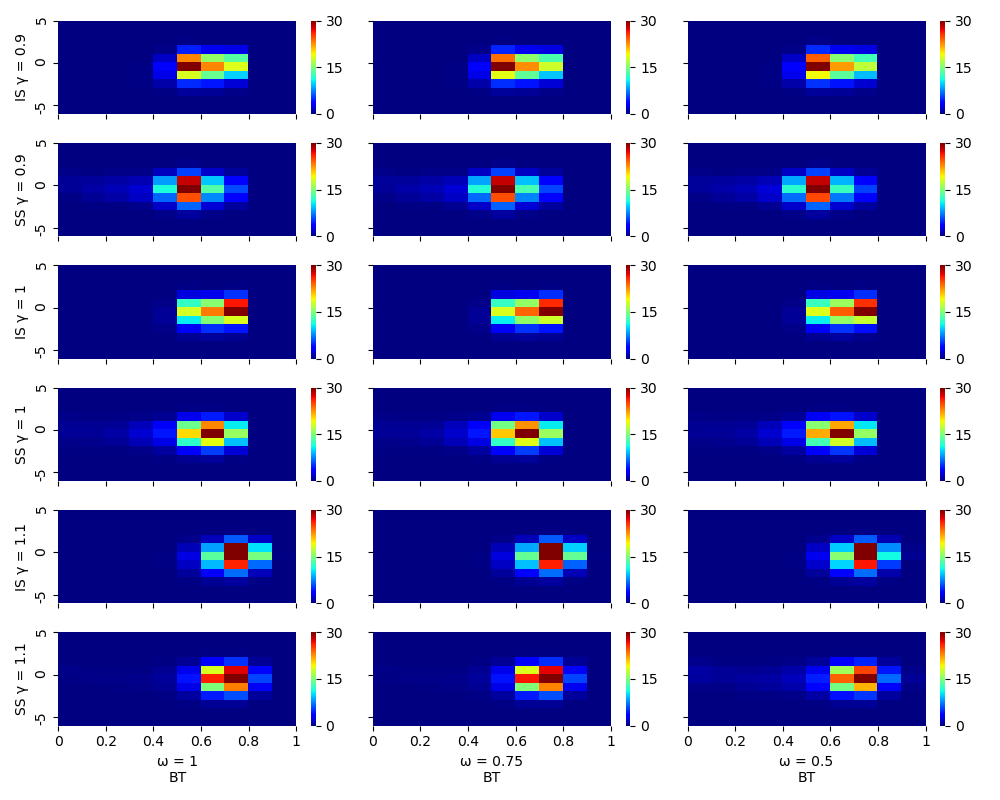


Figure 3: Cartographic profile found using different gamma and omega values during the multilayer community detection. Cartographic profiles are shown as the mean across all participants. Colour bars represent percentage of time spent in a WT / BT bin. WT = module degree z-score, BT = participation coefficient.

It was tested whether the distribution of average (mean across all participants) cartographic profile distribution would change using different gamma and omega values in the multilayer community detection algorithm (Jutla et al., 2011; Mucha et al., 2010) (Figure 3). A gamma and omega values did not appear to greatly affect cartographic profile distribution.


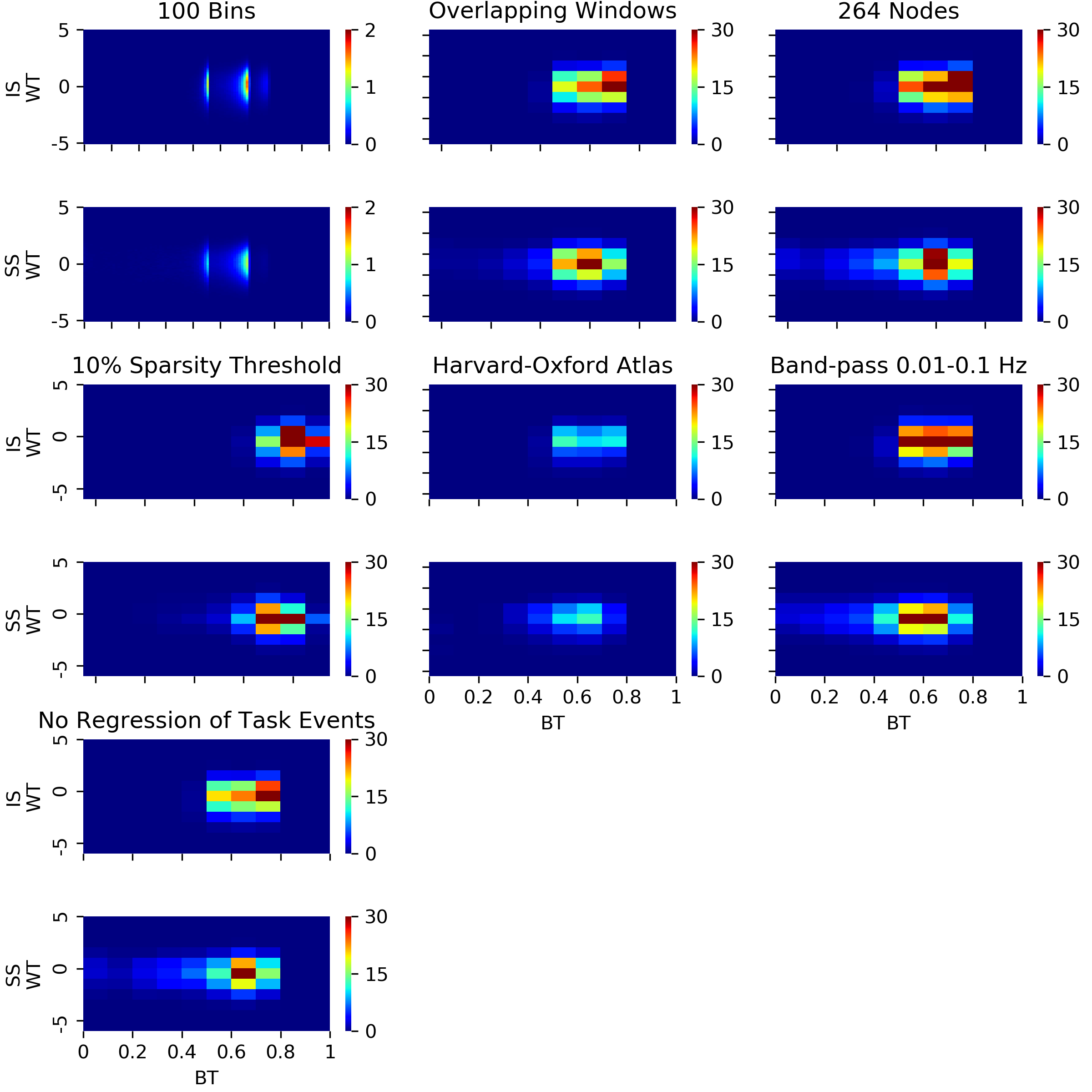


Figure 4: Cartographic profile found using: 100 bins, overlapping windows (164 windows, 1TR steps), 264 nodes from the Power et al. (2011) atlas, a 10% sparsity threshold on windowed functional connectivity matrices, the Harvard-Oxford subcortical / cortical parcellation (Desikan et al., 2006), a band-pass filter of 0.01-0.1 Hz, and no regression of task events. Cartographic profiles are shown as the mean across all participants. Colour bars represent percentage of time spent in a WT / BT bin. WT = module degree z-score, BT = participation coefficient.

A number of analysis choices were also tested to see their effect on the resulting cartographic profile, taken as an average over all participants (figure 4). The use of 100 bins, a 10% sparsity threshold, and an alternative atlas appeared to have problematic effects on the cartographic profile distribution.

## K Mean Clustering – Finding the Optimal Number of Clusters

Previous studies have used K = 2 in the cartographic profiling procedure (Fukushima et al., 2018; Shine, Bissett, et al., 2016; Shine, Koyejo, et al., 2016). To validate this choice, in the present study the optimal K was found by choosing K with the highest average silhouette score (individually for each participant and each of the 3 runs) over a range of K = 2-6. This was computed with the evalclusters() function in Matlab. It was shown that K = 2 was most frequently chosen as the optimal number of clusters (Figure 5).


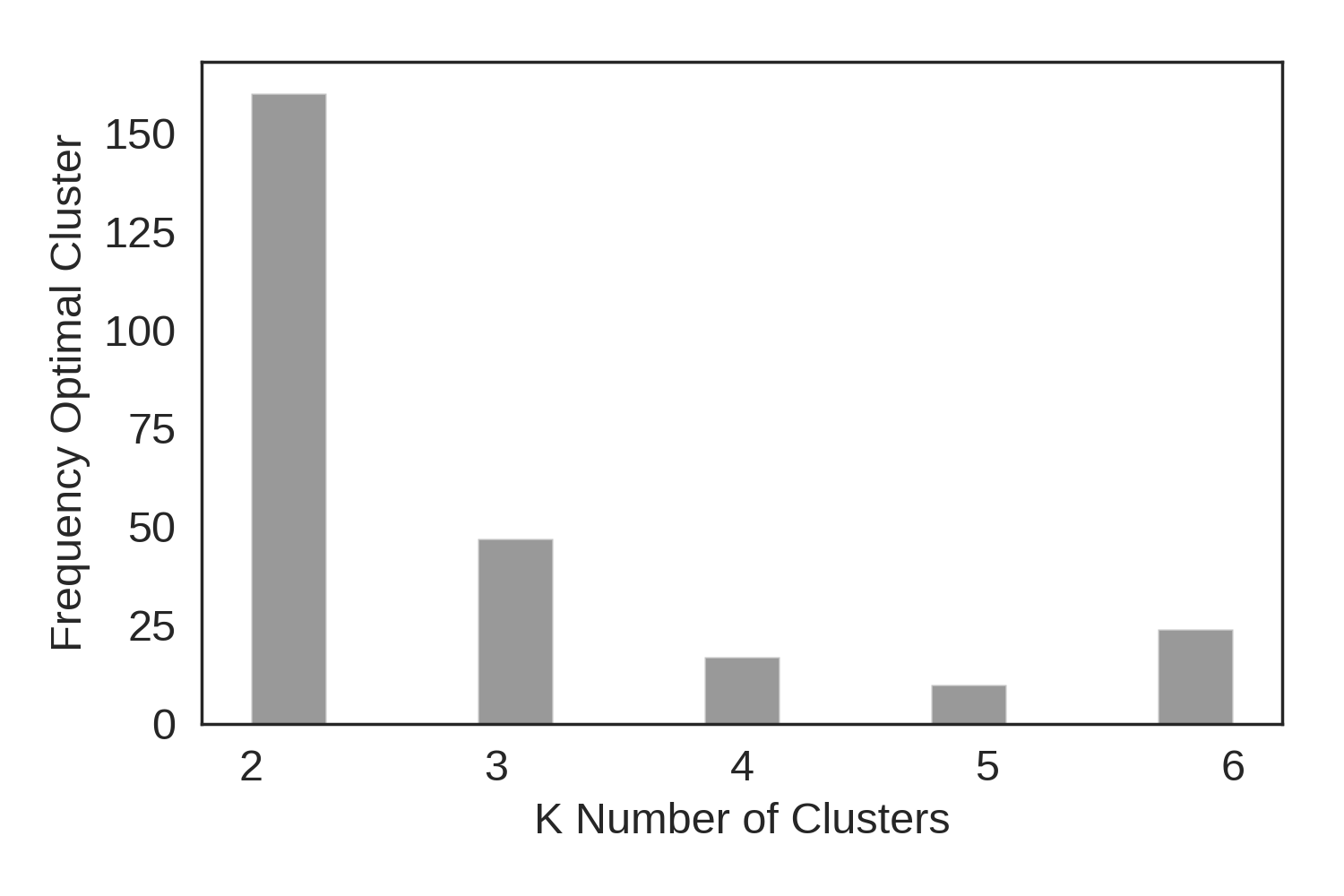


Figure 5: Frequency of each K number clustering solution being chosen as optimal using the average silhouette score.

Though k = 2 was the best cluster solution across participants, k > 2 appeared the ideal clustering solution in some individual cases. In order quantify and control for instances where k = 2 was not the ideal cluster solution the proportion of times k = 2 was chosen using the above method was taken for each participant over 3 runs and 100 iterations. This proportion was entered as a covariate of no interest in the NBS procedure. Using NBS to search for sub-networks related to change in PANSS positive symptoms whilst including this new covariate gave similar results to the main study findings (Figure 6).


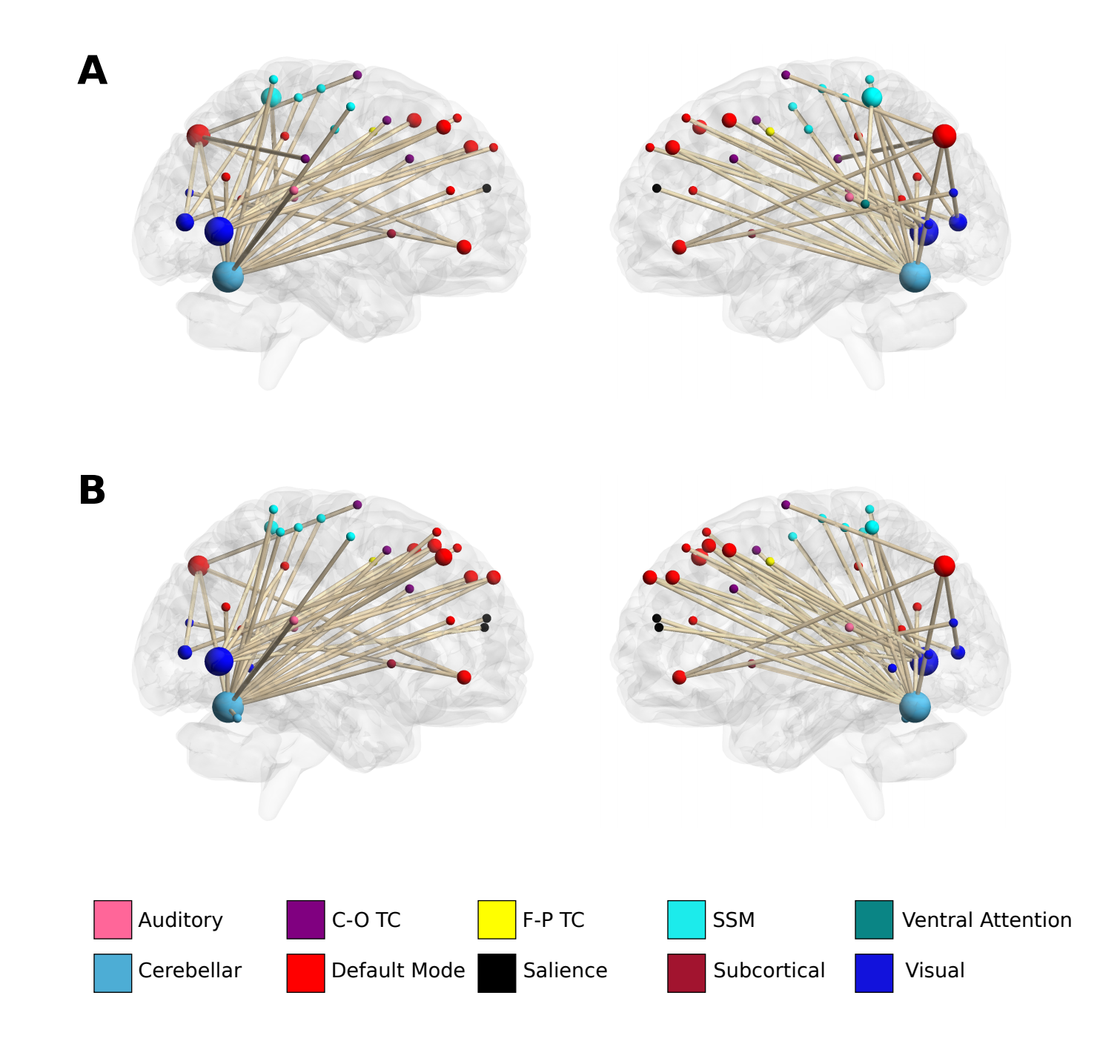
Figure 6: Brain networks showing a significant subnetwork (p < 0.025) associated with change in PANSS positive symptom scores from the NBS procedure (multiple linear regression, 5000 permutations, extent threshold f = 19). A) Subnetwork found using all participants with PANSS follow up scores. B) Results from the same model as A) but including as a covariate the proportion of times k = 2 was the appropriate cluster solution according to Silhouette scores tested across k = 2-6.

## Investigating the Effect Of Window Offset, Length, and Cartographic Profile Resolution

The current study differed from previous studies in using non-overlapping sliding time windows and a 10 x 10 resolution for modelling the CP. Previous studies have used overlapping windows (1 TR offset) and a 100 x 100 resolution (Fukushima et al., 2018; Shine, Bissett, et al., 2016; Shine, Koyejo, et al., 2016). In addition, it is possible different window lengths could affect how effective the CP procedure was. In order to explore window overlap, length, and CP resolution we used the proportional difference of whole brain participation coefficient between integrated (IS) and segregated (SS) states (IS / IS + SS) as a measure of how well the CP procedure performed. If working correctly the CP procedure should produce a set of integrated metastates with a higher participation coefficient. It appeared the choice of non-overlapping windows, a window length of 15 TRs, and a 10x10 CP resolution was optimal for creating a set of integrated metastates (Figures 7).


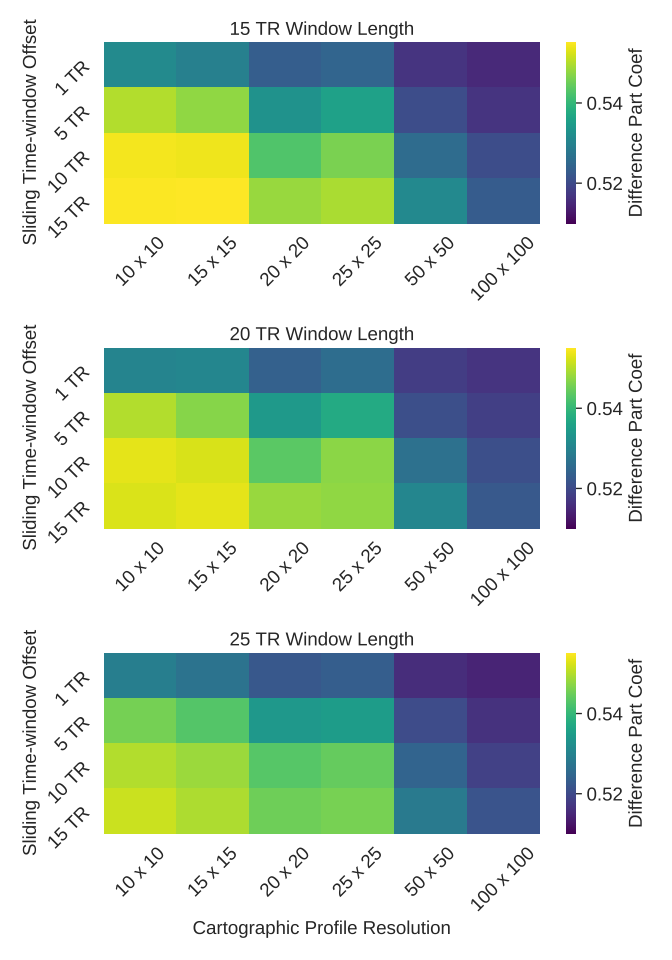
Figure 7: Heat-maps showing proportional difference in whole brain participation coefficient (IS / IS + SS) at a range of sliding time window offsets, cartographic profile resolutions, and window lengths (shown in separate panels). Whole brain participation coefficient was averaged over all participants and 50 iterations.

## Investigating the Effect Of Medication Use on Sub-networks Associated with Change in PANSS Positive Symptom Scores

In order to see whether results were affected by medication use the analysis was repeated excluding those taking antipsychotic medication at baseline and follow up. Results are shown below in Figure 8. Excluding those taking medication at the time of MRI or follow up suggested sub-networks with similar high degree nodes to be associated with change in positive psychosis symptomatology.


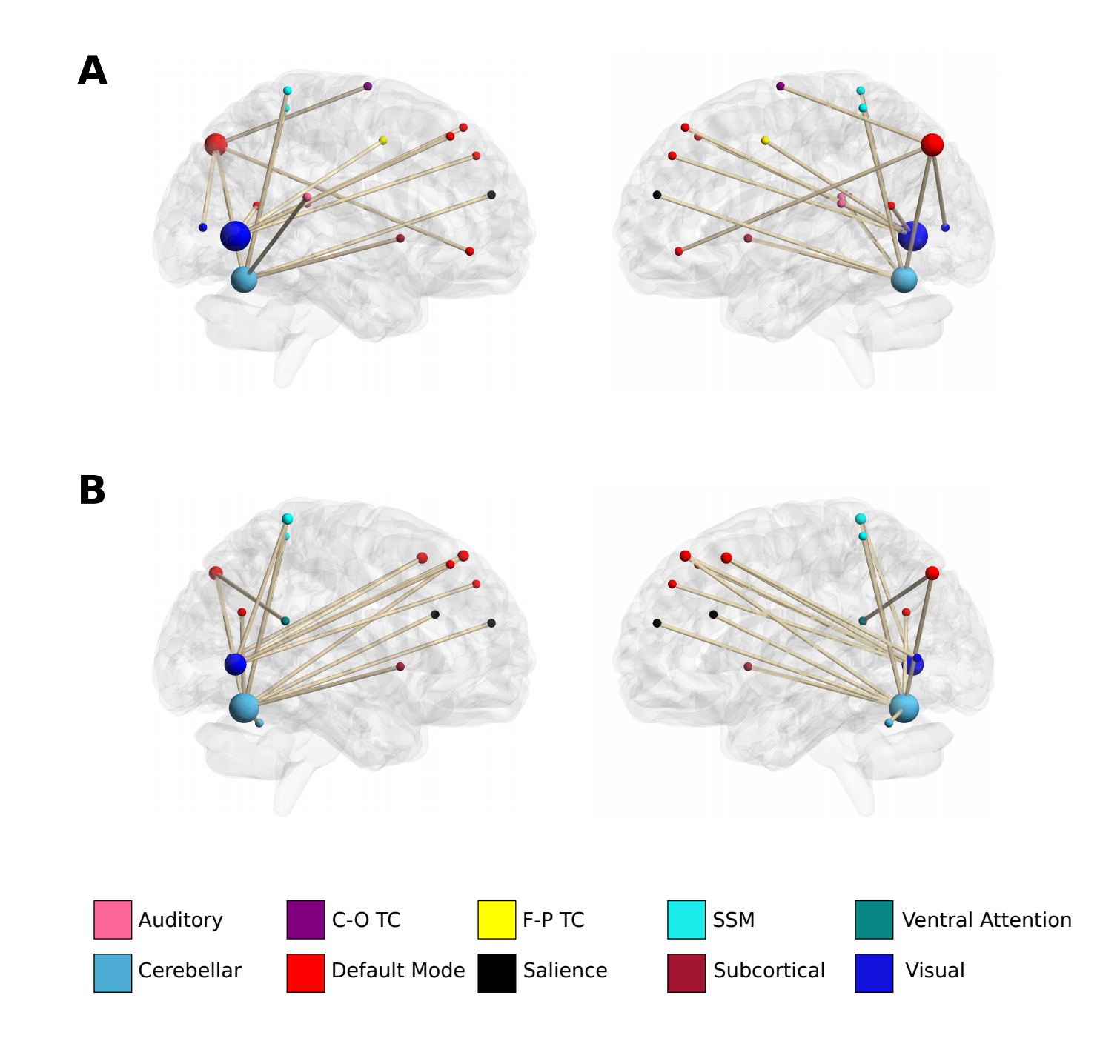
Figure 8: Brain networks showing a significant subnetwork (p < 0.05) associated with change in PANSS positive symptom scores from the NBS procedure (multiple linear regression, 5000 permutations, extent threshold f = 23). A) Subnetwork found using all participants with PANSS follow up scores B) Subnetwork found excluding those who were taking antipsychotic medication at the time of the baseline MRI scan or at the follow up time point.

## Regional Variation in Integration and Segregation

It may be the case that using a framework of global integration and segregation, such as the cartographic profile, disregards local variation in integration / segregation. Node-wise measures of the dynamic engagement of brain areas within and between pre-defined functional networks were therefore computed, and used in between / within group comparisons. This was done using the Recruitment Coefficient (R_i_) and Integration Coefficient (I_i_) as suggested by Mattar et al. (2015). R_i_ indicates a dynamic tendency for a node to share communities with nodes of the same pre-defined network (segregation). I_i_ indicates a dynamic tendency of a node to share communities with nodes of different pre-defined networks (integration) (networks predefined by Power et al. (2011)). These measures require the computation of a node x node module allegiance matrix, in which each cell represents the percentage of times pairs of nodes share the same community over a sequence of community assignments. R_i_ = the mean of within network module allegiance values and I_i_ = the mean of out of network module allegiance values. A module allegiance matrix averaged over the entire sample is shown in Figure 9 and displays typical modular structure with higher module allegiance within typically segregated networks such as the default mode and visual systems.

T values showing the effect of group status on R_i_ and I_i_ values whilst controlling for age, mean FD, antipsychotic use history, and antidepressant use history were computed. Multiple comparisons corrections were computed across all 204 nodes (FDR alpha < 0.05). No regions were significantly different between CHR and HC groups in terms of R_i_ or I_i_. Plotting T values did however show a global trend for higher I_i_ within the CHR group (Figure 10). Notably, in the main study analysis CHR participants spent more time in an integrated metastate defined using the cartographic profiling procedure (mean (SD) % time in integrated state CHR = 63.76 (10.68), HC = 61.05 (9.23). Across the whole sample time spent in an integrated metastate correlated strongly with whole brain I_i_ (R = 0.76).

1. T values showing the association between R_i_ and I_i_ values and PANSS positive symptom scores in CHR participants, whilst controlling for sex, age, mean FD, antipsychotic use history, antidepressant use history, and the number of days to follow up were computed. There were no significant associations between of R_i_ or I_i_ with positive psychosis symptoms (FDR alpha < 0.05).


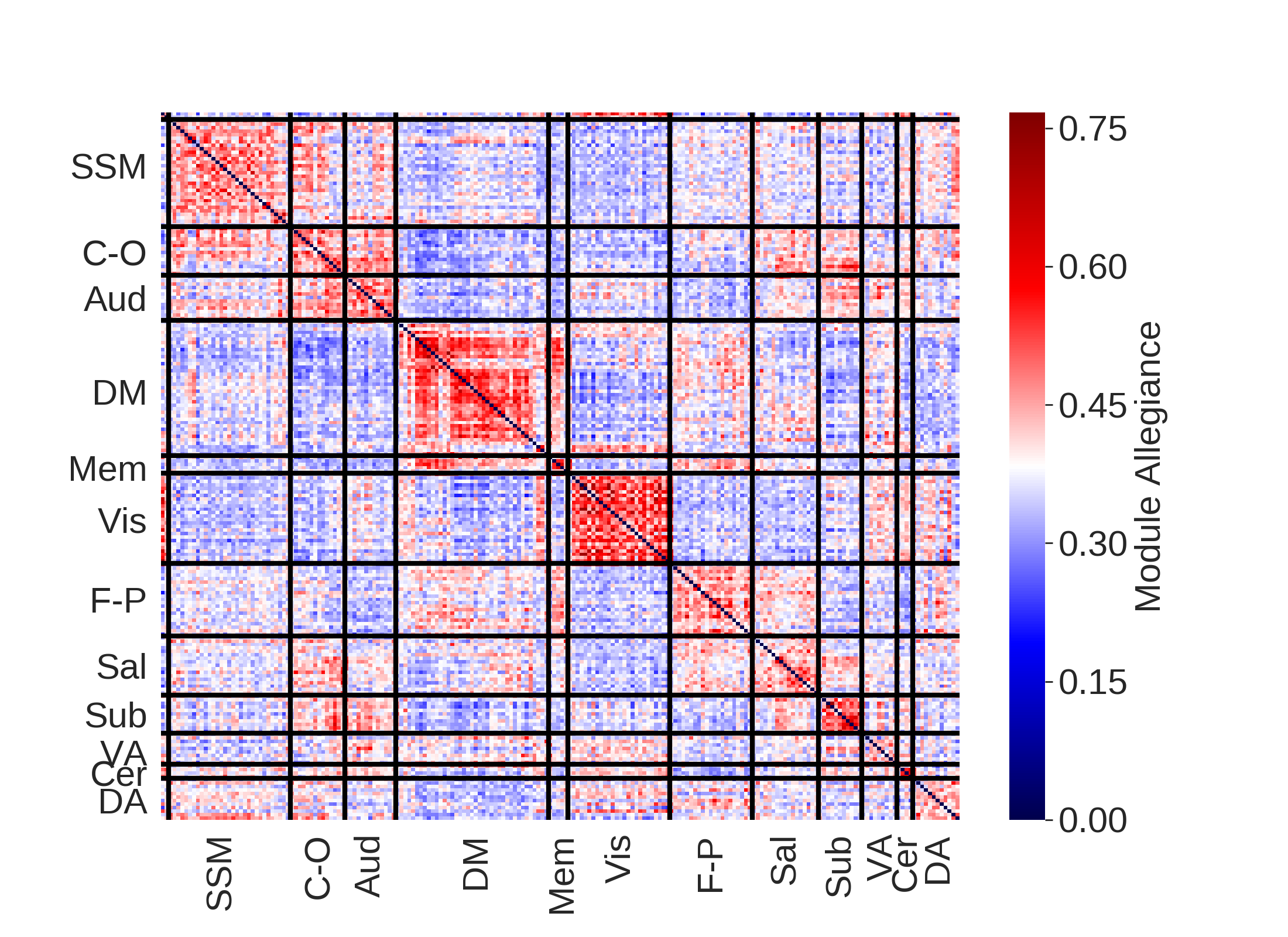
Figure 9: Module allegiance matrix. Module allegiance vales indicate the percentage of time each node pair shared the same time resolved community, averaged over 3 runs and 100 iterations. Abbreviations: SSM: SomatoSensory Motor; C-O: cingular-Opercular Task Control; Aud: Auditory; DM: Default Mode; Mem: Memory; Vis: Visual; F-P: Fronto-Parietal Task Control; Sal: Salience; Sub: Subcortical; VA: Ventral Attention; Cer: Cerebellar; DA: Dorsal Attention.


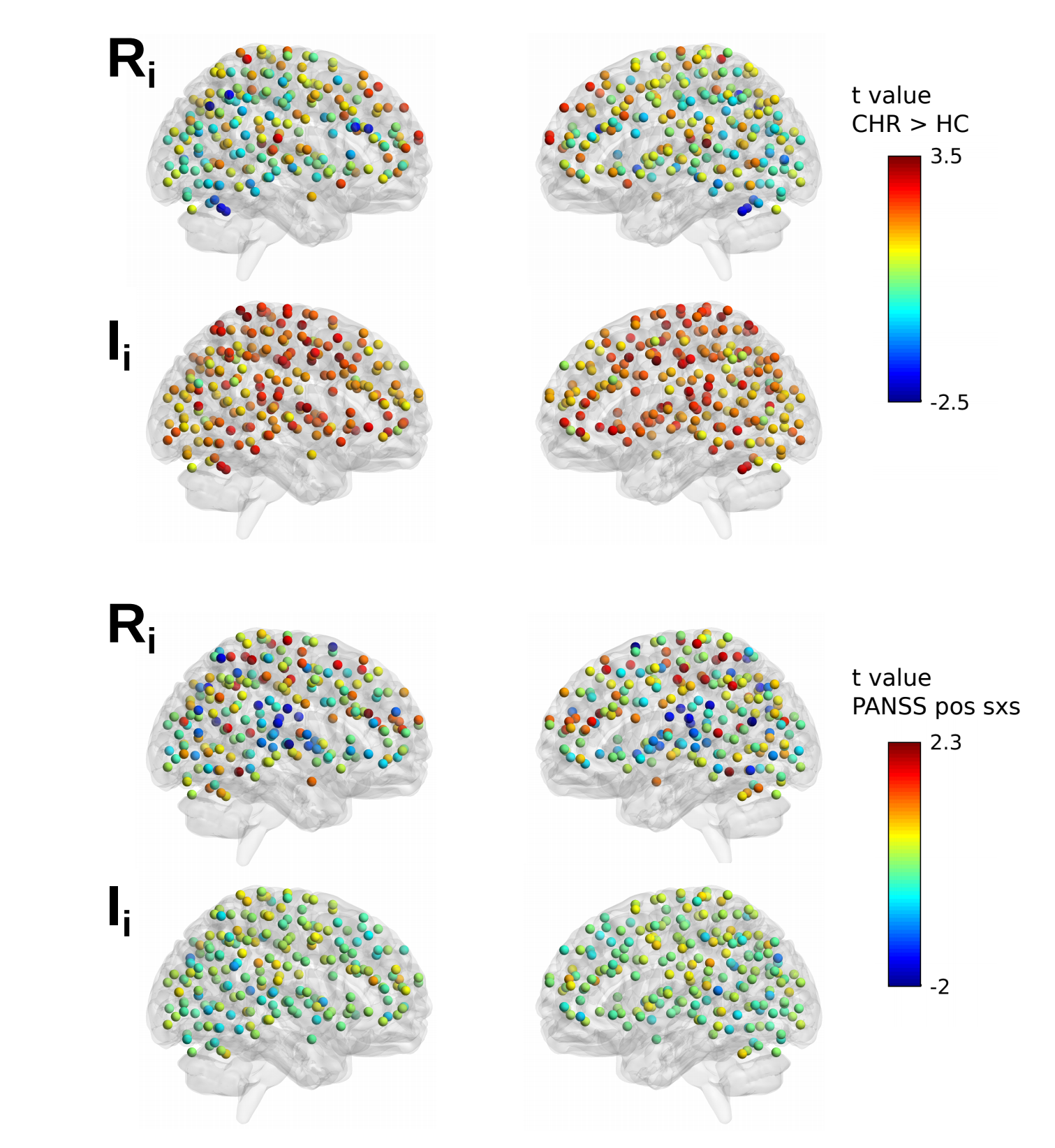
Figure 10: T values from models testing group differences (CHR > HC) and associations with PANSS positive symptom scores in terms of the *Recruitment Coefficient (R_i_) and Integration Coefficient (I_i_). PANSS pos sxs: PANSS positive symptoms.*

# References

Abraham, A., Pedregosa, F., Eickenberg, M., Gervais, P., Mueller, A., Kossaifi, J., Gramfort, A., Thirion, B., & Varoquaux, G. (2014). Machine learning for neuroimaging with scikit-learn. *Frontiers in Neuroinformatics*, *8*, 14.

Avants, B. B., Epstein, C. L., Grossman, M., & Gee, J. C. (2008). Symmetric diffeomorphic image registration with cross-correlation: Evaluating automated labeling of elderly and neurodegenerative brain. *Medical Image Analysis*, *12*(1), 26–41.

Behzadi, Y., Restom, K., Liau, J., & Liu, T. T. (2007). A component based noise correction method (CompCor) for BOLD and perfusion based fMRI. *Neuroimage*, *37*(1), 90–101.

Cox, R. W. (1996). AFNI: software for analysis and visualization of functional magnetic resonance neuroimages. *Computers and Biomedical Research*, *29*(3), 162–173.

Desikan, R. S., Ségonne, F., Fischl, B., Quinn, B. T., Dickerson, B. C., Blacker, D., Buckner, R. L., Dale, A. M., Maguire, R. P., & Hyman, B. T. (2006). An automated labeling system for subdividing the human cerebral cortex on MRI scans into gyral based regions of interest. *Neuroimage*, *31*(3), 968–980.

Esteban, O., Markiewicz, C. J., Blair, R. W., Moodie, C. A., Isik, A. I., Erramuzpe, A., Kent, J. D., Goncalves, M., DuPre, E., & Snyder, M. (2019). fMRIPrep: A robust preprocessing pipeline for functional MRI. *Nature Methods*, *16*(1), 111.

Fonov, V. S., Evans, A. C., McKinstry, R. C., Almli, C., & Collins, D. (2009). Unbiased nonlinear average age-appropriate brain templates from birth to adulthood. *NeuroImage*, *47*, S102.

Fukushima, M., Betzel, R. F., He, Y., van den Heuvel, M. P., Zuo, X.-N., & Sporns, O. (2018). Structure–function relationships during segregated and integrated network states of human brain functional connectivity. *Brain Structure and Function*, *223*(3), 1091–1106. https://doi.org/10.1007/s00429-017-1539-3

Gorgolewski, K., Burns, C. D., Madison, C., Clark, D., Halchenko, Y. O., Waskom, M. L., & Ghosh, S. S. (2011). Nipype: A flexible, lightweight and extensible neuroimaging data processing framework in python. *Frontiers in Neuroinformatics*, *5*, 13.

Greve, D. N., & Fischl, B. (2009). Accurate and robust brain image alignment using boundary-based registration. *Neuroimage*, *48*(1), 63–72.

Jenkinson, M., Bannister, P., Brady, M., & Smith, S. (2002). Improved optimization for the robust and accurate linear registration and motion correction of brain images. *Neuroimage*, *17*(2), 825–841.

Jutla, I. S., Jeub, L. G. S., & Mucha, P. J. (2011). A generalized Louvain method for community detection implemented in MATLAB. *URL Http://Netwiki. Amath. Unc. Edu/GenLouvain*.

Mattar, M. G., Cole, M. W., Thompson-Schill, S. L., & Bassett, D. S. (2015). A Functional Cartography of Cognitive Systems. *PLoS Computational Biology*, *11*(12). https://doi.org/10.1371/journal.pcbi.1004533

Mucha, P. J., Richardson, T., Macon, K., Porter, M. A., & Onnela, J.-P. (2010). Community structure in time-dependent, multiscale, and multiplex networks. *Science*, *328*(5980), 876–878.

Power, J. D., Cohen, A. L., Nelson, S. M., Wig, G. S., Barnes, K. A., Church, J. A., Vogel, A. C., Laumann, T. O., Miezin, F. M., & Schlaggar, B. L. (2011). Functional network organization of the human brain. *Neuron*, *72*(4), 665–678.

Power, J. D., Mitra, A., Laumann, T. O., Snyder, A. Z., Schlaggar, B. L., & Petersen, S. E. (2014). Methods to detect, characterize, and remove motion artifact in resting state fMRI. *Neuroimage*, *84*, 320–341.

Pruim, R. H., Mennes, M., van Rooij, D., Llera, A., Buitelaar, J. K., & Beckmann, C. F. (2015). ICA-AROMA: A robust ICA-based strategy for removing motion artifacts from fMRI data. *Neuroimage*, *112*, 267–277.

Satterthwaite, T. D., Wolf, D. H., Loughead, J., Ruparel, K., Elliott, M. A., Hakonarson, H., Gur, R. C., & Gur, R. E. (2012). Impact of in-scanner head motion on multiple measures of functional connectivity: Relevance for studies of neurodevelopment in youth. *NeuroImage*, *60*(1), 623–632. https://doi.org/10.1016/j.neuroimage.2011.12.063

Shine, J. M., Bissett, P. G., Bell, P. T., Koyejo, O., Balsters, J. H., Gorgolewski, K. J., Moodie, C. A., & Poldrack, R. A. (2016). The dynamics of functional brain networks: Integrated network states during cognitive task performance. *Neuron*, *92*(2), 544–554.

Shine, J. M., Koyejo, O., & Poldrack, R. A. (2016). Temporal metastates are associated with differential patterns of time-resolved connectivity, network topology, and attention. *Proceedings of the National Academy of Sciences*, *113*(35), 9888–9891.

Tustison, N. J., Avants, B. B., Cook, P. A., Zheng, Y., Egan, A., Yushkevich, P. A., & Gee, J. C. (2010). N4ITK: improved N3 bias correction. *IEEE Transactions on Medical Imaging*, *29*(6), 1310.

Zhang, Y., Brady, M., & Smith, S. (2001). Segmentation of brain MR images through a hidden Markov random field model and the expectation-maximization algorithm. *IEEE Transactions on Medical Imaging*, *20*(1), 45–57.
